# Editorial: Mitochondrial Dysfunction in Stroke

**DOI:** 10.3389/fnagi.2022.888952

**Published:** 2022-03-29

**Authors:** Feng Yan, Hailiang Tang, Lin Wang, Lei Huang, John Zhang

**Affiliations:** ^1^Second Affiliated Hospital, School of Medicine, Zhejiang University, Hangzhou, China; ^2^Department of Neurosurgery, Huashan Hospital, Fudan University, Shanghai, China; ^3^Department of Neurosurgery, Loma Linda University, Loma Linda, CA, United States; ^4^Department of Physiology and Pharmacology, Loma Linda University, Loma Linda, CA, United States

**Keywords:** mitochondrial dysfunction, stroke, mitochondrial dynamics, mitophagy and apoptosis, mitochondrial transfer

## Introduction

**Figure 1:**
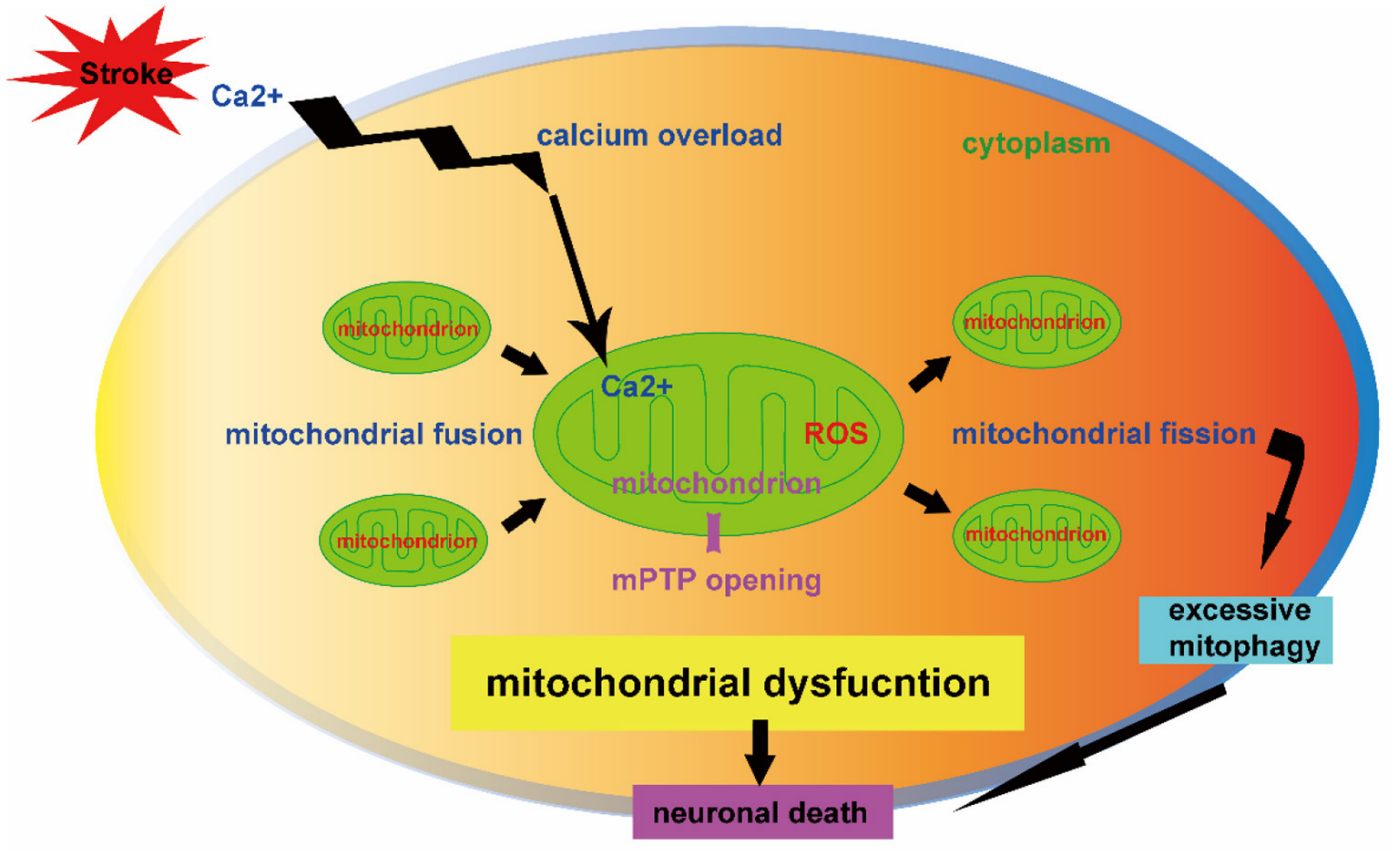
Mitochondrial dysfunction contributing to neuronal death after stroke. The mitochondrial dynamics (fission and fusion) and mitophagy-mediated mitochondrial quality control are key mechanisms in regulation of neuron fate after stroke. The mitochondrial calcium overload, opening of mitochondrial permeability transition pore (mPTP) and over production of reactive oxygen species (ROS) impair mitochondrial dynamics. The effect of mitophagy depends on its injury severity in which overactive mitophagy is detrimental to neuronal survival. The dysregulation of mitochondrial dynamics and excessive mitophagy result in neuronal death.

Stroke is one of the main causes of mortality and remains the second leading cause of death worldwide (Chen et al., 2020). The current therapies of tissue plasminogen activator (tPA) thrombosis and mechanical thrombectomy for ischemic stroke are limited by the narrow therapeutic time window (Zhao et al., 2022). Effective pharmacologic treatments for hemorrhagic stroke are lacking. Emerging evidences demonstrate the importance of mitochondria hemostasis in cell survival and the critical role of mitochondrial dysfunction in the stroke pathogenesis (Kaur and Sharma, 2022). The calcium overload, opening of mitochondrial permeability transition pore (mPTP), and excessive generation of reactive oxygen species (ROS) are mitochondrial pathology contributing to neuronal death after stroke (Figure 1). A better understanding of mitochondrial self-regulation mechanisms and its interaction with other intracellular organelles may reveal novel molecular targets of neuroprotection against stroke (Jia et al., 2021).

In this Research Topic of **“mitochondrial dysfunction in stroke,”** we totally collected 11 articles, majority of which are focusing on the pathophysiology and molecular mechanisms of mitochondrial dysfunction after stroke, as well as potential stroke therapeutics from the mitochondrial perspective.

## Mitochondrial Dynamics and Mitochondrial Dysfunction in Stroke

Mitochondrial dynamics including fusion, fission, selective degradation, and transport processes are important for immunity, apoptosis, and the cell cycle (An et al., 2021; Carinci et al., 2021; Wu et al., 2021; Yang et al., 2022). Zhou et al. reviewed the molecular mechanisms of mitochondrial dynamics and its role in ischemic stroke. The inhibition of excessive mitochondrial fission and preservation of mitochondrial hemostasis are suggested to be potential strategies of neural repair after ischemic stroke.

Mitophagy is a kind of autophagy by specific wipe out of damaged or dysfunctional mitochondria, in order to prevent excessive generation of ROS and neural cell death (He et al., 2021). Mitochondrial fission and mitophagy are two cellular mechanisms that coordinately control mitochondrial quality. In another review article, Shen et al. discussed the involvement of mitochondria dynamics and mitophagy regulation in the pathophysiology of ischemic stroke and ischemic/reperfusion (I/R) injury in particular. The mitophagy-targeted interventions may be potentially applied as an adjunctive therapeutic to extend neuroprotective time window after ischemic stroke. Lei et al.

further reviewed the current research advances of mitophagy regulatory mechanisms after ischemic and hemorrhagic stroke. However, they stressed that the cytoprotective effects of mitophagy modulation in cerebral stroke need further validation by clarifying its possible side effects.

Mitochondrial dynamics can affect energy metabolism and post-stroke neuronal function by regulating the number, morphology, and function of mitochondria. Irisin, a cleaved version of fibronectin domain-containing protein 5 (FNDC5), has been shown to regulate mitochondrial homeostasis. In a mouse model of subarachnoid hemorrhage (SAH), Tu et al. explored the protective effects of irisin and the underlying mechanisms related to mitochondrial biogenesis. The administration of exogenous irisin conserved the mitochondrial morphology and promoted mitochondrial biogenesis, partly through mitochondrial uncoupling protein-2.

Given that early surgical clearance of hematomas does not improve the prognosis in intracerebral hemorrhage (ICH) patients, interventions that attenuate ICH-induced secondary brain injury (SBI) are critical. Chen W et al. summarized the mitochondrial mechanisms in ICH pathology. Abnormal regulation of mitochondrial dynamics that shifts to excessive fission is involved in the pathological process of SBI. Therefore, mitochondrial protection could be a therapeutic target for SBI following ICH.

## Intercellular Mitochondrial Transfer in Stroke

Damaged cells can produce phosphatidylserine, inducing the tunneling nanotubes (TNTs) formation promoting mitochondrial transfer. Intercellular mitochondrial transfer between different cell types as a potential therapeutic approach has been widely studied (Norat et al., 2020; Gomzikova et al., 2021; Lu et al., 2021). Transient focal cerebral ischemia in mice induced astrocytic mitochondria entry to adjacent neurons that amplified cell survival signals (Hayakawa et al., 2016). Mitochondrial transfer improves functional neuron damage after stroke. Gao et al. evaluated the mitochondria transfer and underlying mechanism involved in the neuron-glia crosstalk in primary cultured mouse cortical neurons subjected to a variety of ischemic related insults. They found that the neuron-derived mitochondria may serve as a “help-me” signal and mediate the neuron-astrocyte crosstalk. Promoting the intercellular mitochondrial transfer by accelerating the neuronal releasing or astrocytic engulfing may serve as a potential therapeutic strategy for the treatment of ischemic stroke in the future.

## Conclusion

Mitochondrial dynamics and mitophagy are of great importance in the mitochondrial quantity and quality control. This Research Topic discusses the role of mitochondria in the process of neuronal injury and protection in stroke, aiming to provide valuable insights in aspect of mitochondrial-targeted stroke therapy. Mitochondrial hemostasis preservation, and intracellular mitochondrial transport have a key function in the protection of neuronal injury after experimental stroke, which need future clinical validation in stroke patients. Basic science research is warranted regarding exact interaction mechanism of mitophagy and mitochondria quality control in contribution to pathological or/and protective effect in stroke. The detailed mechanisms of mitochondria release and receptor recognition in donor cells are also needs further investigation in the setting of stroke.

## Author Contributions

FY and HT wrote the editorial equally. LW draw the picture. LH and JZ revised the editorial. All authors contributed to the article and approved the submitted version.

## Conflict of Interest

The authors declare that the research was conducted in the absence of any commercial or financial relationships that could be construed as a potential conflict of interest.

## Publisher's Note

All claims expressed in this article are solely those of the authors and do not necessarily represent those of their affiliated organizations, or those of the publisher, the editors and the reviewers. Any product that may be evaluated in this article, or claim that may be made by its manufacturer, is not guaranteed or endorsed by the publisher.
